# Trends in erectile dysfunction research from 2008 to 2018: a bibliometric analysis

**DOI:** 10.1038/s41443-019-0161-8

**Published:** 2019-06-24

**Authors:** Jialiang Hui, Shuhua He, Ruiyu Liu, Qinyu Zeng, Haibo Zhang, Anyang Wei

**Affiliations:** Department of Urology, Nanfang Hospital, Southern Medical University, 510515 Guangzhou, China

**Keywords:** Sexual dysfunction, Erectile dysfunction

## Abstract

Insufficient penile erection to facilitate vaginal penetration is a medical condition referred to as erectile dysfunction (ED). By the year 2025, the number of ED cases across the world is expected to reach 322 million. There are numerous publications and studies in the field of ED over the past decades. Our aim is to comprehensively analyze the global scientific outputs of ED research and show the trends and hotspots in ED research. Data of publications were downloaded from the Web of Science Core Collection. We used CiteSpace IV and Excel 2016 to analyze literature information, including journals, countries/regions, institutes, authors, citation reports, and research frontiers. Until October 26, 2018, a total of 8880 papers in ED research were identified as published between 2008 and 2018. *Journal of Sexual Medicine* published the most articles. The United States contributed the most publications and occupied leading positions in H-index value and the number of ESI top papers. Maggi M owned the highest co-citations. The keyword “Oxidative stress” ranked first in the research front-line. The amount of articles published in ED research has been stable from 2008 to 2018. The United States showed enormous progress in ED research, and is still the dominant country. Oxidative stress, vardenafil, and late-onset hypogonadism were the latest research frontiers and should be paid more attention.

## Introduction

Insufficient penile erection to facilitate vaginal penetration is a medical condition referred to as erectile dysfunction (ED) [1, 2]. Its diagnosis dates back to over 5000 years ago based on ancient Egyptian literature [3, 4]. The pervasiveness of erectile dysfunction shows a gradual increase in an age-dependent manner according to a cross-sectional and community-based investigation [5]. Moreover, it was found that the prevalence level of severe and moderate ED was 5 and 17%, respectively, in men of 40–49 age-bracket while in men aged 70–79 years they were 15 and 34%, in that order. By the year 2025, the number of ED cases across the world is expected to reach 322 million [6, 7].

There are numerous publications and studies in the field of ED over the past decades. But there is no systematic analysis of the publications. To analyze the progress of events in a given area, bibliometric analysis has been developed as it encompasses quantitative measurements utilizing both statistical and geometrical methods [8, 9]. The bibliometric analyses are primarily directed toward the metrological features of data concerning a specific subject [10], which is often applied in assessing the developments within the field in an extended period. Over the past decades, there have been many bibliometric publications in top medical journals [11–16]. The publishing industry has also broadened its scope beyond researched-based publications to include bibliometric analysis [17].

## Materials and methods

### Sources of data and searching strategies

The articles were retrieved on the same day from the Science Citation Index-Expanded (SCI-E) of the Web of Science Core Collection (WoSCC) on October 26, 2018, to prevent biases due to the daily databases updates. MeSH was used to select the following search terms: = “(TS = (Erectile Dysfunction) OR TS = (Dysfunction, Erectile) OR TS = (Male Sexual Impotence) OR TS = (Impotence, Male Sexual) OR TS = (Sexual Impotence, Male) OR TS = (Male Impotence) OR TS = (Impotence, Male) OR TS = (Impotence)) AND LANGUAGE:(English) Refined by DOCUMENT TYPES: (Article OR Review) Timespan: 2008–2018. Indexes: SCI-EXPANDED”. Review and original research were included in this study.

### Data collection

Two authors working independently downloaded and screened the raw data from WoSCC. The data were analyzed using the CiteSpace IV (Drexel University, Philadelphia, PA, USA) and Excel 2016 (Redmond, WA, USA). In cases where there were discrepancies, they were resolved through discussions.

### Data analysis and statistics

The printing features such as the H-index, impact factor, some annual publications, journal sources, citation counts, journal sources, authors, institutes, and countries/regions, were analyzed using the WoSCC literature analysis wire. The impact factor represents the annual citations received for all the articles published by the journal in a given year [18]. Hence, it provides an estimation of the amount of research data when performing bibliometric analyses. The H-index incorporates the citation impact per publication of institute, country, productivity, etc., and the productivity [19]. This makes it a good tool for measuring the quality of research data.

To study the associations among journals, assess the collaborating teams among countries, institutes, authors, construct visualization maps, capture keywords with strong citation bursts, and identify co-cited authors/references, we used the CiteSpace IV. In this work, 50 most highly cited papers in a one-year slice were used in the individual networks [20–24]. The term frequency-inverse document frequency (TF-IDF) weighting was utilized for the analysis of data in each group. TF-IDF is a statistical algorithm reflecting how necessary a word to a corpus of documents [25].

## Results

### Growth forecast and yearly publications

**Fig. 1:**
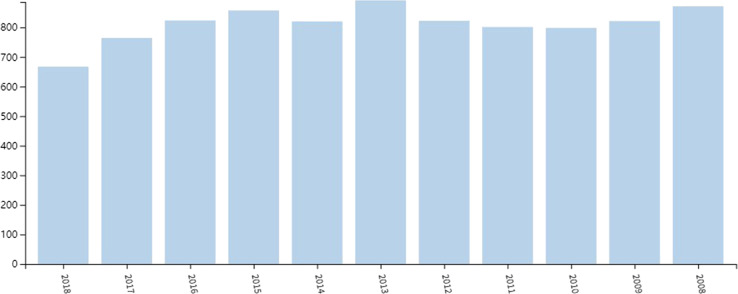
Publication outputs. The annual number of publication in erectile dysfunction research from 2008 to 2018

In total, 8880 articles matched the retrieval criteria. Figure 1 shows the total articles published in each year, with the trend ranging from 866 papers in 2008 to 759 papers in 2017. On average, there were 822 articles per year, and the number of publications per year remained stable. As of October 26, 2018, 662 articles were published in 2018.

### Journal distribution

With regard to publications in the ED subject, there were 1587 academic journals publishing in this subject and Table 1 shows the top 20 journals. *Journal of Sexual Medicine* (impact factor (IF) 2017 = 3.339) had the highest publications (1495 articles, 17.724%), followed by *International Journal of Impotence Research* (IF 2017 = 1.517; 385 publications; 4.564%), *BJU International* (IF 2017 = 4.688; 293 publications; 3.474%), *Urology* (IF 2017 = 2.300; 249 publications; 2.952%), and *Journal of Urology* (IF 2017 = 5.381; 214 publications; 2.537%).

**Table 1:** The top 20 journals that published articles in erectile dysfunction research

Rank	Journal title	Country	Count	Percent	IF 2017
1	Journal of Sexual Medicine	USA	1495	17.724	3.339
2	International Journal of Impotence Research	England	385	4.564	1.517
3	BJU International	England	293	3.474	4.688
4	Urology	USA	249	2.952	2.300
5	Journal of Urology	USA	214	2.537	5.381
6	Asian Journal of Andrology	China Mainland	186	2.205	3.259
7	Andrologia	Germany	169	2.004	1.588
8	European Urology	Netherlands	116	1.375	17.581
9	Andrology	USA	108	1.280	2.734
10	Aging Male	England	106	1.257	2.500
11	PLOS One	USA	95	1.126	2.766
12	Journal of Andrology	USA	79	0.937	2.473 (2014)
13	International Journal of Clinical Practice	England	76	0.901	2.000
14	International Urology and Nephrology	Netherlands	71	0.842	1.692
15	Sexual Medicine	England	67	0.794	1.457
16	Urologia Internationalis	Switzerland	62	0.735	1.508
17	International Journal of Urology	Japan	59	0.699	1.941
18	International Braz J Urol	Brazil	57	0.676	0.976
19	World Journal of Urology	USA	50	0.593	2.981
20	Journal of Mens Health	Ireland	46	0.545	0.231

**Fig. 2:**
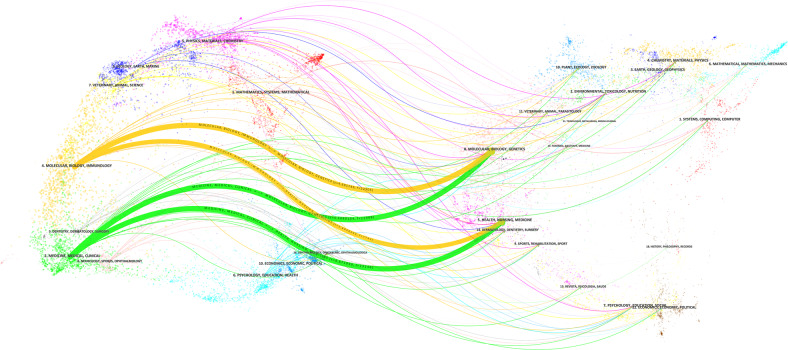
The dual-map overlay of journals related to erectile dysfunction research. There were four citation paths. The top yellow path, papers published in molecular/biology/immunology journals mostly cited journals in molecular/biology/genetics area; the middle green path, papers published in molecular/biology/immunology journals partially cited journals in health/nursing/medicine area; the middle green path, papers published in medicine/medical/clinical journals partially cited journals in molecular/biology/genetics area; the bottom green path, articles published in medicine/medical/clinical journals partially cited journals in health/nursing/medicine area

Figure 2 displays the dual-map overlay of journals. The right and left side resembled the citing and cited journals maps, individually. The labels showed the description of fields covered by the journal. The lines on the map, originated from the left to the right, served the citation links. There were four main citation paths on the map.

### Profiling of institutes and countries

**Fig. 3:**
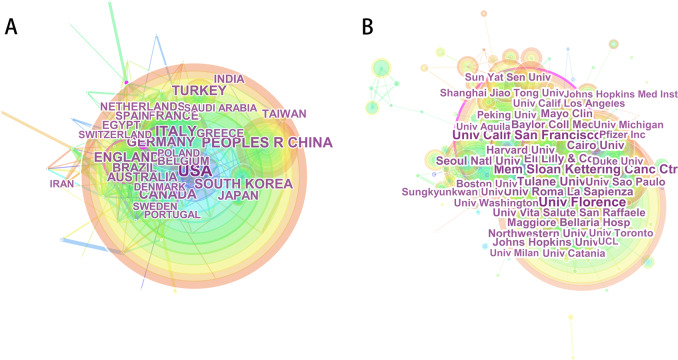
The distribution of countries and institutes. **a** The network map of countries/regions that in the case of erectile dysfunction (ED) research. **b** The network map of institutes involved in erectile dysfunction research

A total of 114 regions/countries published 8880 articles in ED research. There were very wide collaborating teams among these regions/countries (Fig. 3a). Among the leading 20 regions/countries (Table 2) engaged in ED research, 2896 publications were from the USA, followed by Italy (985), Peoples R China (867), England (639), and Turkey (530).

**Table 2:** The top 20 countries and institutes publications in erectile dysfunction research

Rank	Country/region	Count	Percent	Institute	Count	Percent
1	USA	2896	32.613	Univ Florence	218	2.455
2	Italy	985	11.092	Univ Calif San Francisco	185	2.083
3	Peoples R China	867	9.764	Mem Sloan Kettering Canc Ctr	161	1.813
4	England	639	7.196	Tulane Univ	127	1.43
5	Turkey	530	5.968	Univ Roma La Sapienza	112	1.261
6	Germany	475	5.349	Cairo Univ	104	1.171
7	South Korea	445	5.011	Eli Lilly Co	103	1.16
8	Canada	427	4.809	Univ Sao Paulo	97	1.092
9	Brazil	405	4.561	Seoul Natl Univ	96	1.081
10	Australia	294	3.311	Univ Vita Salute San Raffaele	94	1.058
11	France	282	3.176	Baylor Coll Med	92	1.036
12	Japan	276	3.108	Maggiore Bellaria Hosp	92	1.036
13	Egypt	244	2.748	Harvard Univ	89	1.002
14	Taiwan	228	2.568	Mayo Clin	86	0.968
15	Spain	221	2.489	Johns Hopkins Univ	85	0.957
16	Netherlands	219	2.466	Northwestern Univ	84	0.946
17	Belgium	172	1.937	Pfizer Inc	81	0.912
18	India	167	1.881	Univ Calif Los Angeles	77	0.867
19	Greece	157	1.768	Univ Washington	75	0.844
20	Sweden	141	1.588	Duke Univ	73	0.822

As shown in the result, about 7449 institutes were engaged in research in the field of ED. Among the institutes, there were extensive collaborations (Fig. 3b). About 36% of the total publications were from the top 20 institutes (Table 2). Univ Florence topped the list, followed by the Univ Calif San Francisco, Mem Sloan Kettering Canc Ctr, Tulane Univ, and Univ Roma La Sapienza.

### Analysis of highly cited, H-index, and average citations

**Fig. 4:**
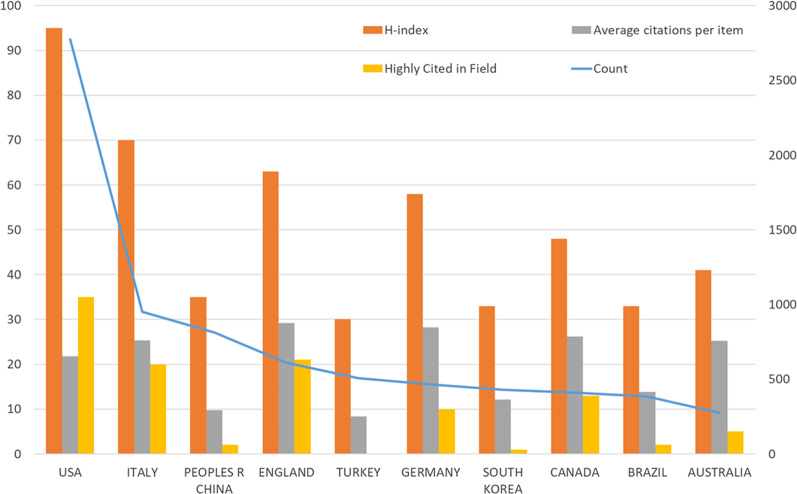
The distribution of highly cited, H-index, and average citations in the top ten countries

Among the top ten rich countries (Fig. 4), the United States contributed the most number of highly cited papers (35) and achieved the highest H-index value (96). Followed by Italy, Peoples R China, England, and Turkey. Out of the figure, France had the highest levels of average citations per item (33.99), but in the overall ranking was the 12th place. Although France is a non-English speaking country, ASSISTANCE PUBLIQUE HOPITAUX PARIS APHP is an important institution in the field of ED research in France. Research support in the field of ED is strong, with more than 40% of research coming from this institution. Also in France, there are more than 4000 researchers working on ED research, and there are 120 articles by GIULIANA FRANCOIS, accounting for 24.2%.

### Profiling of authors

**Fig. 5:**
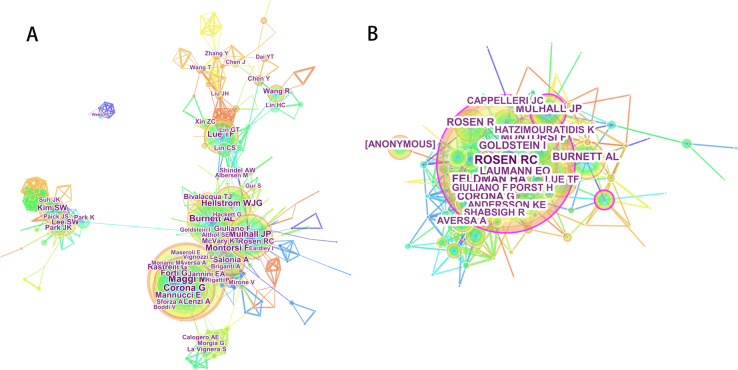
The distribution of authors. **a** The network map of active authors offered to erectile dysfunction research. **b** The network map of co-cited authors offered to erectile dysfunction research

As shown in the result, 28,321 authors were engaged in ED research. The collaboration network among the authors is shown in Fig. 5a. Of the top 20 contributing authors (Table 3), Maggi M (with 172 articles) was ranked first, followed by Corona G (139 publications), Burnett AL (114 publications), Mulhall JP (104 publications), and Montorsi F (96 publications).

**Table 3:** The top 20 authors, co-cited authors, and co-cited references in erectile dysfunction research

Rank	Author	Count	Co-cited author	Count	Co-cited reference	Count
1	Maggi M	172	Rosen RC	2407	HATZIMOURATIDIS K, 2010, EUR UROL, V57, P804	277
2	Corona G	139	Feldman HA	1107	THOMPSON IM, 2005, JAMA-J AM MED ASSOC, V294, P2996	221
3	Burnett AL	114	Corona G	940	BHASIN S, 2010, J CLIN ENDOCR METAB, V95, P2536	172
4	Mulhall JP	104	Montorsi F	922	INMAN BA, 2009, MAYO CLIN PROC, V84, P108	161
5	Montorsi F	96	Mulhall JP	756	LEWIS RW, 2010, J SEX MED, V7, P1598	158
6	Hellstrom WJG	92	Burnett AL	753	SANDA MG, 2008, NEW ENGL J MED, V358, P1250	149
7	Mannucci E	81	Rosen R	727	WU FCW, 2010, NEW ENGL J MED, V363, P123	142
8	Lue TF	80	Laumann EO	682	SHAMLOUL R, 2013, LANCET, V381, P153	142
9	Forti G	79	Goldstein I	674	ROSEN R, 2003, EUR UROL, V44, P637	140
10	Salonia A	72	Lue TF	657	SELVIN E, 2007, AM J MED, V120, P151	135
11	Kim SW	68	Porst H	570	MCVARY KT, 2007, J UROLOGY, V177, P1071	126
12	Rosen RC	66	Giuliano F	567	MONTORSI F, 2008, EUR UROL, V54, P924	121
13	Lee SW	64	Cappelleri JC	562	ESPOSITO K, 2004, JAMA-J AM MED ASSOC, V291, P2978	118
14	Rastrelli G	64	[anonymous]	546	GRATZKE C, 2010, J SEX MED, V7, P445	107
15	Bivalacqua TJ	59	Andersson KE	545	MALAVIGE LS, 2009, J SEX MED, V6, P1232	103
16	lenzi A	57	Shabsigh R	534	LINDAU ST, 2007, NEW ENGL J MED, V357, P762	100
17	Giuliano F	55	Hatzimouratidis K	519	MCVARY KT, 2007, J UROLOGY, V177, P1401	99
18	Jannini EA	52	Aversa A	505	DONG JY, 2011, J AM COLL CARDIOL, V58, P1378	98
19	Mcvary KT	52	Althof SE	477	GAZZARUSO C, 2008, J AM COLL CARDIOL, V51, P2040	96
20	Wang R	52	Bivalacqua TJ	472	JACKSON G, 2006, J SEX MED, V3, P28	95

The data of author citations were analyzed using CiteSpace and visualized in a co-citation network (Fig. 5b). For the top 20 co-cited authors (Table 3), Rosen RC (2407 co-citations) was ranked first, followed by Feldman HA (1107 co-citations), Corona G (940 co-citations), Montorsi F (922 co-citations), and Mulhall JP (756 co-citations).

### Analysis of references

**Fig. 6:**
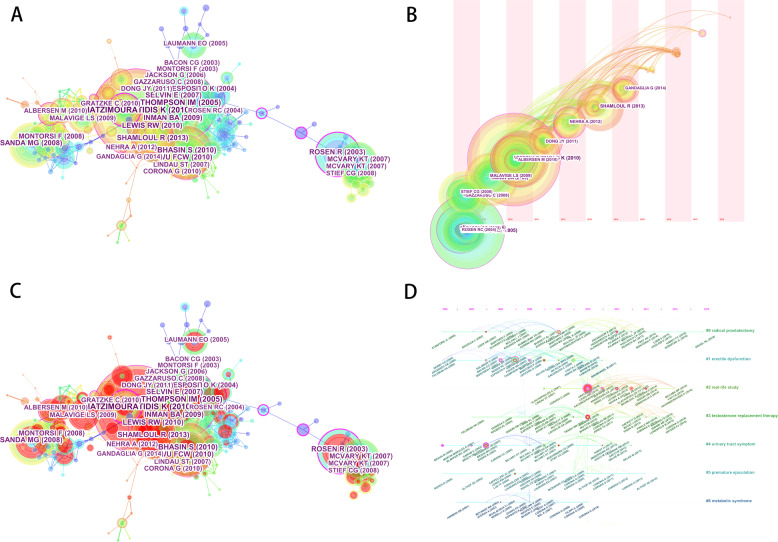
The analysis of references. **a** The co-citation map of references from publications in erectile dysfunction (ED) research. **b** The timeline view of co-cited references from publications in ED research. **c** The burst words map of references from publications in ED research. **d** The timeline view of burst words from publications in ED research

To construct a network of co-cited references, the CiteSpace IV was used, and the results are shown in Fig. 6a. As shown in the figure, studies of HATZIMOURATIDIS (2010) [26], THOMPSON (2005) [27], BHASIN (2010) [28], INMAN (2009) [29], and LEWIS (2010) [30] are the most frequently cited documents (all more than 150 times). These may be the focus of the ED field research and the classic literature, which researchers should pay more attention. Furthermore, Fig. 6c highlights the burst and key literature in the references to present the results (Fig. 6c) better. The terms extracted from the reference list of the articles were used to the name the groups as shown in Fig. 6d. In the network (Fig. 6d), the top4 bulky clusters were named “#0 radical prostatectomy”, “#1 erectile dysfunction”, and “#2 real-life study”, and “#3 testosterone replacement therapy”, respectively. Moreover, Fig. 6b, d showed the timeline trajectory of the clusters.

### Analysis of burst keywords

**Fig. 7:**
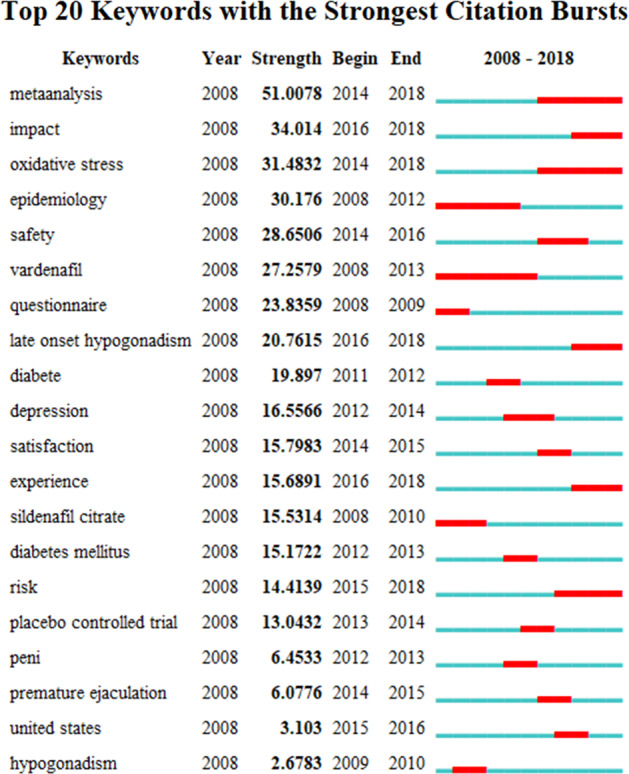
The keywords with strong citation bursts in articles related to erectile dysfunction research published from 2008 to 2018

The CiteSpace IV tool was used to burst to keywords that showed a high-citation burst (Fig. 7). Exclude keywords that are not research priorities, hose that had citation bursts from 2008 onwards were: “oxidative stress” (2014–2018), “vardenafil” (2008–2013), “late-onset hypogonadism” (2016–2018), “diabetes” (2011–2012), and “sildenafil citrate” (2008–2010) (excluding some keywords that are not directly related to research).

## Discussion

### General information

The aim of this study was to comprehensively analyze the global scientific outputs of ED research in 2008–2018, and show the trends and hotspots in ED research. Based on the analysis, the publication trend showed a stable trajectory within the search period. To obtain enough number of publications, the time range of publications was set from 2008 at identifying them. We limited the period to the publications made after 2008 to obtain enough number of publications. With regard to the top 20 academic journals, one journals, *European Urology* (IF 2017 = 17.581) had an IF higher than 10; one other, *Journal of Urology* (IF 2017 = 5.381) had an IF between 10 and 5; three journals, *BJU International* (IF 2017 = 4.688), *Journal of Sexual Medicine* (IF 2017 = 3.339), and *Asian Journal of Andrology* (IF 2017 = 3.259) had an IF between 5 and 3.

The number of publications and citations was highest in the USA compared with the top 20 contributing regions/countries in ED frontier studies. A similar performance was noted about the top H-index and ESI articles as well as the quality of research quality, in which the USA ranked first.

About 3277 papers (23.994%) belonged to the top 20 institutes. Similarly, three out of the top five institutes were from the USA and two from Italy.

### Citation analysis

Each author in the top 20 groups had more than 52 publications which were considered to the frontiers in ED research. However, only eight of these authors were listed in the top co-cited authors’ list, implying that high-ranking researchers should focus on the quality of research in addition to the number of papers.

As shown in Fig. 6b, analysis of the timeline view of the co-citation clusters showed that the highly co-cited articles were obtained within 2008–2014. Twenty-nine items were identified between 2008 and 2014 from the top 50 co-cited articles retrieved using CiteSpace IV. Hence, this time-frame can be regarded as “golden period” in the ED research in the last decade. The top 20 co-cited articles are shown in Table 3. A paper published by Hatzimouratidis K in 2010 received the highest number of citations, seconded by 221 co-citations for a paper by Thompson IM in 2005, Bhasin S (2010, 172 co-citations), Inman BA (2009, 161 co-citations), and Lewis RW (2010, 158 co-citations), who published papers in *Jama-J Am Med Assoc*, *J Clin Endocr Metab*, *Mayo Clin Proc*, and *J Sex Med*. Additional high-impact factor has generated more articles on ED research in the last decade, e.g., *Lancet* (five articles) and the *New England Journal of Medicine* (four articles). These journals contributed significantly to the ED field.

### Research frontiers

The burst keywords were identified using the CiteSpace IV, which may be used to predict research hinterlands. In Fig. 7, the red lines represented the period of citation bursts, while blue lines represent the time interval. The following list contains the three frontiers of ED research:

#### I. Oxidative stress

Compared with the general population, the occurrence of ED in men with diabetes is about threefold higher [31, 32]. Many factors are associated with the occurrence of ED, including neurological insults and vascular as well as other metabolic disorders [33]. Chronically atmospheric glucose collections were found to increase the production of nitrogen species (RNS) and reactive oxygen (ROS), which in turn causes glycation end‐products (AGE) [34]. Following phenomenon, alterations in the levels of endothelial or neuronal‐derived nitric oxide (NO) may compromise the vasorelaxation mechanisms in the diabetic corpus cavernosum [35, 36]. Decreased levels of NO is thought to cause a low response of diabetic men with ED to oral phosphodiesterase‐5 inhibitor therapies [37]. Apart from the alterations to penile vasorelaxation, elevated ROS decreases the levels of antioxidants and lipid peroxidation which leads to damages the DNA and eventually the cavernosal cells structures and functions [38, 39]. Several studies had provided evidence that ROS exerted detrimental effects on ED. The observations have demonstrated that the application of antioxidants increases endothelial NO synthase (eNOS) and reduces superoxide formation (O2−) thereby improving diabetic ED [38, 40–42].

#### II. vardenafil: phosphodiesterase-5

**Phosphodiesterase-5** (PDE-5) is a cyclic guanosine monophosphate (cGMP)-specific phosphodiesterase (PDE) that is highly expressed in the penile corpus cavernosum, and PDE-5 inhibitors, such as Vardenafil, are used to treat ED. Vardenafil highly specific and potent PDE-5 inhibitor which is used to treat ED. Its mechanism of action involves smooth muscle relaxation, which is a critical factor for penile erection [43]. Vardenafil is 5–10 fold potent compared with sildenafil, another typical PDE-5 inhibitor [44]. It is well-tolerated, and has an excellent safety profile and few adverse effects. However, some side-effects such as nasal congestion, indigestion, flushing, and headache are associated with its use [45, 46]. Vardenafil has a rapid mode of action, and therefore ED patients achieve sufficient erection within 10–15 min following drug intake [47, 48]. During its hepatic metabolism, 14 metabolites are formed, of which N-desethyl vardenafil (M1) is the most predominant, and it has been found to be pharmacologically active [49, 50].

#### III. Late-onset hypogonadism

Serum testosterone levels decrease in men with aging at the rate of 1–2% per year after the age of 40. The clinical condition of testosterone decreased with specific symptoms has been widely accepted as late-onset hypogonadism (LOH) syndrome [51]. The Massachusetts Male Aging Study demonstrated that prevalence of LOH syndrome in men between the ages of 40 and 69 ranged from 6.0 to 12.3% and 2.4 million men in the United States were estimated to be affected [52]. Widely recognized clinical signs of LOH syndrome include ED, decreases in muscle mass and strength, anemia, obesity, osteoporosis, depression, and deterioration of insulin resistance [53–55]. Therefore, LOH syndrome is a clinical condition that could affect the function of multiple organ systems, and LOH syndrome itself is an essential sign of many other potentially dangerous states. Testosterone replacement therapy (TRT) is a widely accepted treatment in the prevention and amelioration of many of the symptoms and conditions associated with LOH syndrome [55].

### Strengths and limitations

To the immeasurable of our knowledge, this study is the first bibliometric analysis on ED research bearing over the past decade. The data analysis manner was relatively physical. Nevertheless, most publications retrieved from the database were written in English, causing incomplete analysis to some extent. Furthermore, this study consisted particularly of original and reviewed articles published between 2008 and 2018 and classified by the Web of Science. It may not be enough to describe all ED literature, such as other record types published in journals, books, and conferences were not included. The analysis in this study was based on articles recorded in the SCI-E of the WoSCC. Each journal to which the SCI-E articles belong had its corresponding citation report provided by the Web of Science. Although other databases such as PubMed, Scopus, and Embase could give a broader range of coverage, much of the “extra coverage” could be credited to journals with potentially limited readers.

Given that our objective was to conduct a high-quality bibliometric analysis to identify research trends in the core of the ED field, the SCI-E articles from WoSCC may be the only suitable choice. Accordingly, the results from other databases were not included. Finally, although the Web of Science database is still updating, this study covers the vast majority of papers in ED research since 2008; new data may not influence the final results.

In conclusion, the number of publications in ED research has been increasing over the past decade. The United States, Italy, and China were the top three countries contributing to ED studies. There were active collaborations between developed countries. The United States was still the autocratic country engaged in ED research.

Rosen RC, Feldman HA, and Corona G may be ideal candidates for academic cooperation. Oxidative stress, vardenafil, late-onset hypogonadism, diabetes, and sildenafil citrate may be hinterlands in this field, and researchers should pay close consideration to relevant studies in the coming years.
